# Novel Reference Method for the Characterization of PD Measuring Systems Using HFCT Sensors

**DOI:** 10.3390/s24123788

**Published:** 2024-06-11

**Authors:** Eduardo Arcones, Fernando Álvarez, Javier Ortego, Fernando Garnacho

**Affiliations:** 1 Department of Ingeniería Eléctrica, Universidad Politécnica de Madrid, Ronda de Valencia 3, 28012 Madrid, Spain; fernando.alvarez@upm.es (F.Á.); javier.ortego@ampacimon.com (J.O.); fernando.garnacho@ffii.es (F.G.); 2 AMPACIMON, C/de la Antracita, 7, Nave 15, 28045 Madrid, Spain

**Keywords:** partial discharges, insulation testing, condition monitoring, performance evaluation, power system modeling, model-driven development, sensor phenomena and characterization

## Abstract

During their lifespan, high-voltage (HV) electrical systems are subjected to operating conditions in which electrical, mechanical, thermal and environmental-related stresses occur. These conditions over time lead to unforeseen failures caused by various types of defects. For this reason, there are several technologies for measuring and monitoring the electrical systems, with the aim of minimizing the number of faults. The early detection of defects, preferably in their incipient state, will enable the necessary corrective actions to be taken in order to avoid unforeseen failures. These failures generally lead to human risks and material damage, lack of power supply and significant economic losses. An efficient maintenance technique for the early detection of defects consists of the supervision of the dielectrics status in the installations by means of on-line partial discharge (PD) measurement. Nowadays, there are numerous systems in the market for the measurement of PD in HV installations. The most efficient with a reasonable cost will be those that offer greater security guarantees and the best positioned in the market. Currently, technology developers and users of PD measuring systems face difficulties related to the lack of reference procedures for their complete characterization and to the technical and economic drawback of performing the characterization tests on site or in laboratory installations. To deal with the previous difficulties, in this paper a novel method for the complete and standardized characterization of PD measuring systems is presented. The applicability of this method is mainly adapted for the characterization of systems operating in on-line applications using high-frequency current transformer (HFCT) sensors. For the appropriate application of the method, an associated and necessary scale modular test platform is used. In the test platform, the real on-site measuring conditions of an HV insulated distribution line are simulated in a controlled way. Practical characterizations, showing the convenience and advantages of applying the method using the modular test platform, are also presented.

## 1. Introduction

For the detection of PD activity various methods have been developed: electromagnetic, acoustic, optical and analysis of chemical by-products [1,2,3,4]. Among these methods, the electromagnetic one is the most used due to its versatility and efficiency [5,6,7]. Electromagnetic signals generated by PD travel long distances in HV installations, therefore by applying an electromagnetic method, e.g., measuring with HFCT sensors, the capability of detecting defects in the monitored installations considerably increases. In order to carry out diagnoses of the dielectrics status, there are various measuring systems in the market based on the electromagnetic detection method [8,9,10]. With the functionalities implemented in these systems, appropriate diagnosis of the dielectrics insulation condition can be achieved. However, the degree of success may vary depending on the measuring system used and on the analyst’s expertise. The functionalities developed for these systems are mainly related to their capability to:

- filter the background electrical noise present in the installations to perform the acquisitions with an adequate sensitivity,
- perform autonomous diagnosis and generate alarms when a critical defect is detected,
- determine the phase or phases affected by the defects,
- discriminate the presence of all the insulation defects present in the supervised installation,
- locate the emplacement of the defects,
- identify the type of defect associated with each PD source,
- and identify the defective elements of the installation.

An adequate characterization of the measuring systems functionalities enables the identification of their strengths and weaknesses. In this respect, currently, technology developers and electrical company’s users of these systems face the following three difficulties.

- Lack of reference or standardized procedures that allow their characterization in a complete and reproducible way.
- Technical and economic difficulty to characterize them in a complete and reproducible way in on-site or laboratory installations. This is due to the lack of availability of these installations, high running costs, restricted use at a single site and the impossibility (in on-site installations) or great difficulty (in laboratory installations) to control the noise conditions.
- With the use of the above installations, with the current state of the art, it is not possible to create the controlled measurement conditions necessary for the complete and reproducible characterization of the systems. Furthermore, with the same installation, it is not possible to make comparisons of results over time in various emplacements for various technologies.

Concerning the first difficulty, although various researchers have presented interesting measuring methods and techniques to perform accurate diagnosis [11,12,13,14], very few studies are focused on a comprehensive characterization of the technical functionalities developed [15,16,17]. Moreover, in none of them a reference method is proposed to be applied for the complete and reproducible characterization of measuring or monitoring systems measuring in real conditions. In this paper a new reference method is presented, showing its use with an associated portable test platform [18] required for its appropriate application. This test platform was developed by the authors to deal with the second and third problems previously indicated. The combination of the method with the associated test platform makes characterization of measuring systems possible at any time, everywhere and in a complete and reproducible way without the requirement of carrying out HV tests in on-site installations or in complex laboratory setups. 

The next section is focused on the explanation of the method and the characterization tests specified in it. For a better understanding of the method application, a case study of a measuring system characterization is presented in Section 3. Lastly, Section 4 is dedicated to the conclusions.

## 2. Characterization Method and Associated Test Platform

The authors of this paper presented in [15,16,17] methods for the evaluation of PD instruments or analyzers that are applied measuring simulated defects in small-scale systems [15,16] or measuring them directly with the acquisition units [15,16,17]. These methods are valid to characterize some measuring instrument or analyzer functionalities. However, if they are used for the complete characterization of the measuring systems, the following shortcomings arise.

- The methods do not consider their applicability in three-phase installations, thus some functionalities as those developed for the identification of the affected phase, or for defect detection analyzing the acquisitions obtained in the three phases, cannot be characterized. In industrial applications, the supervision is carried out simultaneously in the three phases, thus making it more effective.
- When the PD pulses used are measured, they are not representative of those acquired in real on-site measuring conditions. Furthermore, in [16] the PDs generated are very close to the measuring point.
- The on-site noise measuring conditions in the sensor environment, when measuring in one or various positions of an installation, are not considered or controlled. Thus, the system’s characterization considering the noise influence cannot be properly performed.
- When tests cells are used for the PD generation [16], HV application is required and the performance of repetitive tests over time is not possible due to the stochastic behavior of the pulses.
- The measuring conditions concerning the sensor coupling and the technical characteristics of the earth connections of the setups differ from those of a real installation [16].
- When the PDs generated with tests cells [16] or an analog generator [15] are measured by the sensors, as the physical characteristics of real on-site installations are not reproduced, the following technical aspects are not properly considered: the phase coupling and the polarity, attenuation, distortion and reflection of the pulses. Thus, the complete and adequate characterization of the systems cannot be performed.
- If the scale systems were not used and the characterizations were performed with an analog generator [15,17,19,20,21,22], injecting the signals that simulate defects and noise conditions directly into the acquisition unit would not be possible to perform the characterizations in a complete way with real sensors in a physical system and measuring in a non-limited number of points.

In order to overcome the previous shortcomings, the novel reference method described in Section 2.2 has been developed. This method comprises an associated physical ad hoc test platform for its applicability [18]. A short description of the test platform is presented in Section 2.1 for a better understanding of the method description and application.

### 2.1. Scale Modular Test Platform

**Figure 1:**
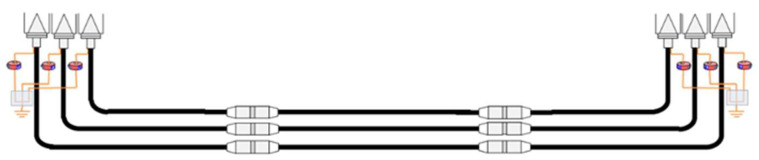
HV distribution system considered for its simulation in the test platform.

For the method application, in a first version and for simplicity, the test platform to be used [18] simulates an HV insulated distribution system with a straight joint configuration, connected at both ends to a GIS substation, see Figure 1. Specifically, the cable system to be simulated is a 66 kV, 1200 mm^2^ aluminum conductor and has 9 mm thick cross-linked polyethylene (XLPE) insulation [18].

**Figure 2:**
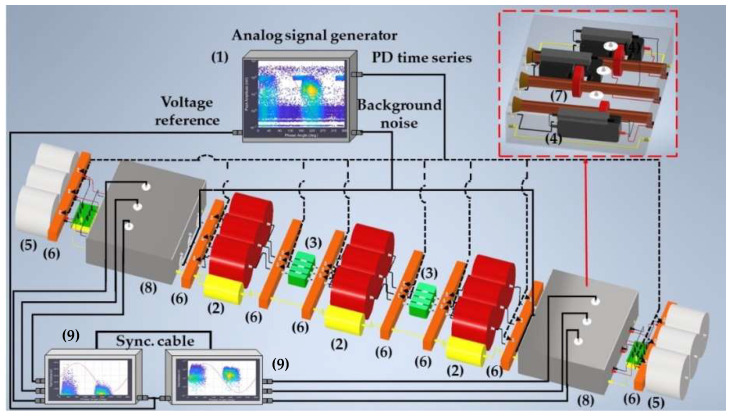
Test platform developed to be used in the characterization of PD measuring systems.

For a proper characterization of the functionalities, the test platform is made up of the following parts (subsystems), which are shown in Figure 2.

- Analog signal generator (ASG) subsystem (1). The signals generated by this element (high-frequency transient PD pulses and electrical noise) are of the same nature as those of real installations.
- Scale module subsystem, consisting of three-phase insulated cable elements (2), straight junction chambers (3), cable–GIS connection elements (4) and GIS modules (5).
- Defect injection subsystem (6) for the simulation of the PD sources. The defects can be simulated in the GIS compartments, cable terminals and cable joints.
- HFCT sensor subsystem (7). The measurements are performed in two positions, at the beginning and at the end of the distribution system.
- Noise injection subsystem (8) for the simulation of the background noise measuring conditions of a real installation in the sensor environment. Within this subsystem are the cable–GIS connection elements (4) and the HFCT sensors (7).
- Measuring subsystem (9), with a three-channel acquisition unit per measuring point.

### 2.2. Characterization Method

**Figure 3:**
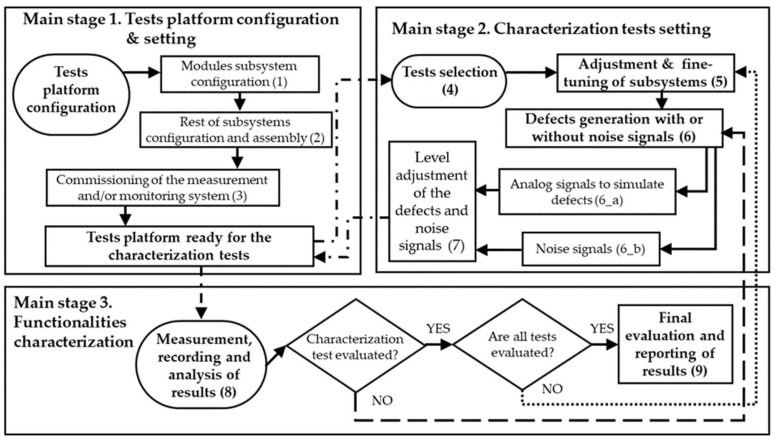
Flowchart of the method for the measuring system’s characterization.

The proposed method is articulated in three main stages, see the flowchart of Figure 3: (Stage 1) test platform configuration and setting, (Stage 2) characterization test setting and (Stage 3) functionalities’ characterization. Its implementation makes the characterization of PD measuring systems feasible in a complete and reproducible way.

In stage 1 of the method, the test platform shown in Figure 2 is configured and set. In this platform the same measuring conditions as those of real installations are simulated due to the design and integration of the first five subsystems indicated in Section 2.1. PD pulses and noise signals measured in real installations have amplitude levels lower than a few units of volts, thus, as the signals to be injected in the test platform are always below 10 volts, the characterizations are performed using the platform without any electrical risk. In step 1, the module subsystem is configured. In step 2, the rest of the subsystems are configured and the set is assembled, including the measurement subsystem. For the functionalities’ characterization, the positioning of the defects and the sensors is performed in strategic points of the test platform through the defect injection subsystem and the sensor subsystem, respectively. In distribution line PD monitoring, non-invasive HFCT sensors are the most frequently used, thus the test platform measuring points are adapted for the installation of this type of sensor. In step 3, the measurement systems are commissioned. After the execution of these three steps, the test platform is ready to perform the characterizations.

In stage 2 the characterization tests are prepared and set. In step 4 a set of tests is considered from those specified in a non-exclusive manner in Table 1. These tests are explained in detail in Section 2.3. In step 5, for each test the subsystems of the test platform are adjusted, considering the type of system to be characterized. The tests are prepared and executed sequentially. To perform the tests, in step 6 isolation defects with or without noise signals are generated in the platform. These defects are simulated by injecting analog signals with impedance matching in the defect injection subsystem (step 6_a). The analog signals are reproduced with the ASG subsystem. 

To simulate the measurement conditions of real installations, it is necessary to create the same noise conditions present in them. This requirement is fulfilled by the following considerations with respect to these two types of noise signals.

- The background random noise is generated with the ASG and injected into the noise injection subsystem (step 6_b), where the same noise conditions of the real installations are simulated in the environment of the sensors [18].
- The pulse-type noise, which propagates in a conducted way, is also generated with the ASG and injected in the same way as the analog signals that simulate the defects (step 6_b).

All the analog signals generated with the ASG are adjustable in waveform, magnitude and frequency spectrum. For a complete characterization of the systems, in step 6, it is possible to generate several coexisting defects in different phases and locations, also superimposing various noise signals. The PD and noise signals generated with the ASG were measured beforehand in real cable–GIS systems and laboratory tests cells, where real insulation defects and noise conditions were present. The measured signals were subsequently treated for their proper generation, considering technical aspects related to the measuring conditions, the type of sensors used, the position of the defects or the frequency intervals of interest. This allows performing the tests on the platform with the same conditions and casuistry as in a real installation.

In step 7, the defects and noise signal levels are adjusted to discrete values simulating various degrees of criticality and interferences, which makes it possible to fully characterize the functionalities by obtaining behavior trends. This requirement of adjusting the defect and noise signal levels is shown in the flowchart of Figure 3 by the feedback loop indicated with the dashed line. In addition, as the tests are performed sequentially, the feedback loop shown with the dotted line has been added. When steps 5, 6 and 7 are performed for each test, the test platform is ready to carry out the measurements with the measuring system to be characterized.

In stage 3 the functionalities’ characterization is carried out sequentially. Thus, in step 8, the measurements are performed. Subsequently, the recorded data are processed and analyzed. Finally, in step 9, the system characterization is carried out and the corresponding results report is drawn up. The interactions between the three stages of the method are indicated in Figure 3 by the dotted and dashed lines. 

It is important to indicate that for the characterization of the measuring systems and to make comparisons between them, all tests must be performed under controlled electrical noise conditions. Furthermore, the base noise must be previously quantified. Thus, it is recommended to perform the characterization tests in shielded laboratories or in anechoic chambers.

### 2.3. Characterization Tests

In this section the tests indicated in Table 1 are described.

**Figure 4:**
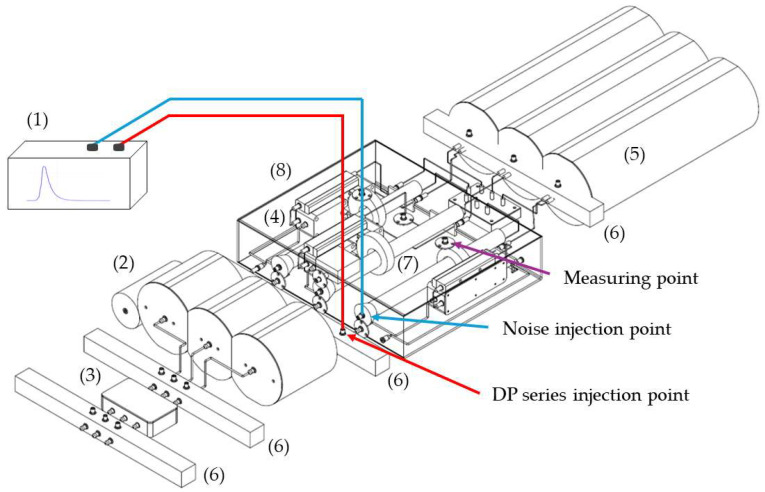
ASG subsystem (1) connected (red line) to an element of the defect injection subsystem (6) and (blue line) to a module of the noise injection subsystem (8), with (2) being a cable element, (3) a straight junction chamber, (4) the cable–GIS connection elements, (5) a GIS module and (7) the sensors subsystem.

**Figure 7:**
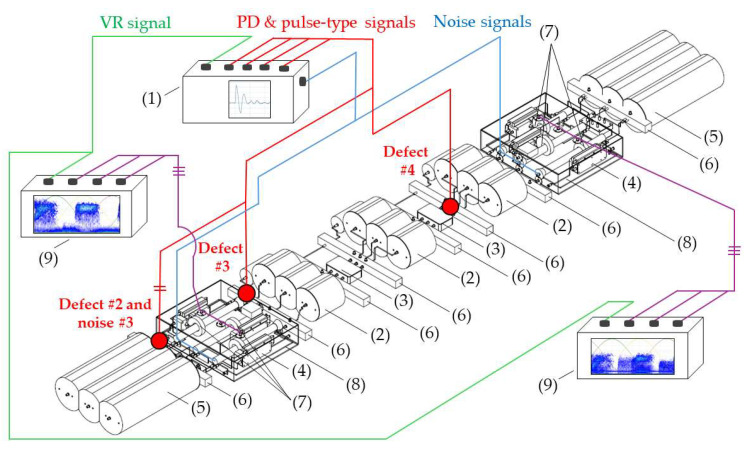
Insulation defects and noise injection in the test platform. (1) ASG subsystem, (2) cable elements, (3) straight junction chambers, (4) cable–GIS connection elements, (5) GIS modules, (6) defect injection subsystem, (7) sensor subsystem, (8) noise injection subsystem and (9) measuring subsystem.

**Test 1. Sensitivity in the detection.** The transient signals generated by PD are of low energy and very short duration. When they are measured with HFCT sensors, the pulses last less than 1 µs and have an amplitude lower than 1 V, making them difficult to detect, especially when on-site measurements are performed. To ensure effective diagnostics, measuring systems must have a good sensitivity to detect these signals. The sensitivity in the detection test proposed is performed by injecting a series of pulses with the ASG subsystem in one phase of the defect injection subsystem element positioned at the end of the line. These pulses are measured with the sensor of the same phase located at that position. Figure 4 shows in detail the elements that make up this part of the platform, the injection point (see the red line) and the measuring point. In this test, the noise signal (blue line in Figure 4) is not injected. The complete setup is shown in Figure 2 and Figure 7.

**Figure 5:**
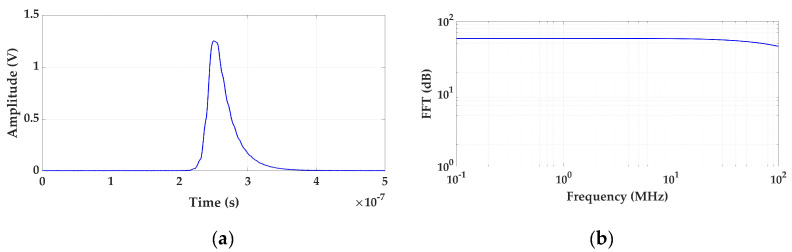
Injected pulse in test 1. (**a**) Waveform of the pulse and (**b**) frequency spectrum.

To characterize the sensitivity, the background base noise signal acquired by the measuring unit is first analyzed in the time and frequency domain and then a calibration is performed. The scale factor is determined injecting PD pulses of 1000 pC. Subsequently, the pulse charge is decreased in successive steps (500 pC, 200 pC, 100 pC, 50 pC, 20 pC and 15 pC). From 15 pC the steps are smaller until the measuring system is no longer sensitive. In each step the PD charge is analyzed and compared to the generated values. To establish a reference measurement, the test must be performed within a frequency range specified in IEC 60270, specifically a bandwidth of fc = 250 kHz ± 150 kHz must be selected. When the measured PD charge differs by more than 15% from the reference values, it is considered that the system is no longer sensitive. To avoid the influence of background noise, the average value of the samples of 500 captures of a pulse, synchronized with the time instant of the pulse generation, can be calculated at each step of charge. In this way, the only prevailing signal is the one of the average pulse, with the background base noise being negligible. The waveform and frequency spectrum of the pulses generated in this test are shown in Figure 5. With this test the scale factor linearity of the analyzer is also checked. An example of this test realization is shown in Section 3.2. For a general overview of the results the use of Table 4 is recommended.

**Test 2. Noise rejection.** When the measurements are performed in on-site installations, background noise conditions can adversely affect PD detection. To cope with this difficulty, PD measuring systems incorporate hardware and software tools to reject the noise. The purpose of this test is to evaluate the noise immunity of the measuring systems. This test is performed by simulating in the test platform an internal defect of a known Q_IEC_ value and PD repetition rate. All the PD series generated have a Q_IEC_ value between 500 pC and 550 pC and a repetition rate between 50 and 60 pulses per period (ppp), see Table 5 of results. The values of the injected series are known only to the evaluator. This internal defect, indicated in Table 2 as #1, is injected and measured in the same way as the pulses of test 1, see Figure 4. 

**Figure 6:**
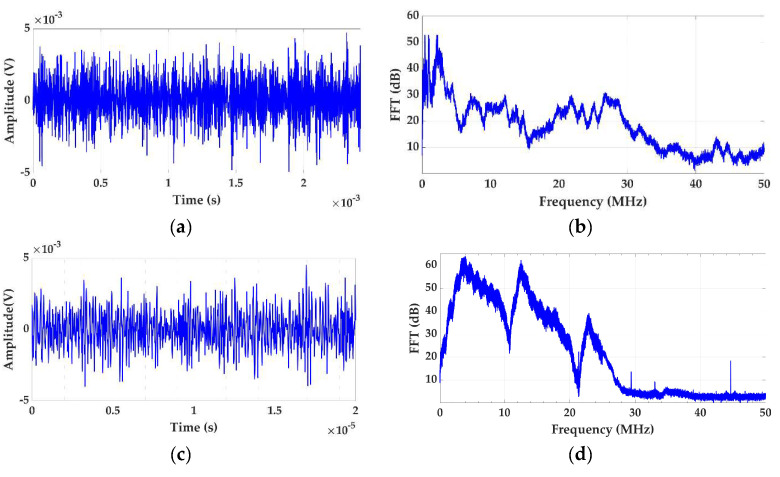
Injected noise signals #1 and #2, (**a**) noise #1 in the time domain, (**b**) noise #1 frequency spectrum, (**c**) noise #2 in the time domain and (**d**) noise #2 frequency spectrum.

In this test, a first measurement is performed by overlapping an on-site aleatory noise signal with an amplitude of 3σ = 3.8 mV on the background base noise. This noise signal, indicated in Table 2 as #1, is shown in Figure 6a,b. The measured noise is analyzed and then the necessary adjustments are made in the hardware and software of the measuring system. Afterwards, the system is calibrated by injecting together with noise #1 the calibration pulses of test 1. Once the system is calibrated, this test can be conducted with the following two levels of difficulty.

- Level 1 (low difficulty). The internal defect #1 is measured simultaneously with the on-site noise #1, which is generated in successive steps with increasing levels of amplitude.
- Level 2 (high difficulty). The internal defect #1 is measured simultaneously with a different on-site aleatory noise. This second noise, indicated in Table 2 as #2, is shown in Figure 6c,d. By generating the noise signal #2, the changing noise conditions of real installations are considered. With this level of difficulty, the noise signal is also generated with increasing levels of amplitude.

With both levels of difficulty, noise signals #1 and #2 are injected in the noise injection subsystem, see in Figure 4 the blue line, with the four amplitude levels indicated in Table 5 (3σ = 3.8 mV, 3σ = 7.6 mV, 3σ = 11.4 mV and 3σ = 15.2 mV). Thus, the PD time series generated are measured superimposed onto the electrical noise signals in the same conditions of a real installation. For each noise level, the charge and PD rate are analyzed and compared to the reference values. When the values of charge and rate obtained differ by more than 75%, it is considered that the measuring system is no longer sensitive enough. A practical case of this test is shown in Section 3.2. For a general overview and analysis of the results the use of Table 5 is recommended. From now on, the adjustments performed in this test and the scaling factor obtained must remain invariable when the rest of the tests are performed.

**Test 3. Autonomous diagnosis and alarm management.** An important functionality of PD measuring systems is related to their capability to automatically process the registered data in order to generate preliminary diagnoses and alarms when critical PD activity is detected. With an automatic processing and a good alarm generation strategy, an optimized supervision of the installations for the prevention of breakdown occurrence is achieved. This is because the intervention of the expertise analyst only occurs when a critical defect is detected. Stand-alone systems enable cost savings and consequently a significant increase in the capacity to supervise the installations. For these reasons, autonomous systems are highly demanded by electrical companies. The automatic diagnosis functionalities are parameterized in the management software by expert technicians or analysts at the beginning of the measurements, considering the characteristics of the installation to be supervised. When PD activity is detected, programmed alarms are triggered depending on the importance of the defects. False alarms should be avoided in order to have proper autonomous processing results. 

In some cases, the alarm management is performed in more than one level [23]. In this regard, the authors suggest that a first level can be defined for certain defect detection conditions. Only when this level is activated is the intervention of an expert analyst required for a complete verification study, where the criticality of the defect is determined in detail. When it is considered that a defect of relative importance, detected at the first level, does not pose a risk in the short term, it is advisable to monitor its trend and program a second level alarm for it. This second level alarm will be triggered when the defect becomes more critical. 

To evaluate the capability of the measuring systems to operate autonomously and their alarm management, the following two tests are proposed.

- Measurement of at least three insulation defects positioned in various elements and locations of the installation and in more than one phase, along with at least one pulse-type noise and one aleatory noise. Subsequent processing and analysis of the registered data in automatic mode. The defects #2, #3 and #4 and the noises #2 and #3 indicated in Table 2 are proposed for this test. The three defects and noise #3 are generated with the ASG subsystem and injected into the defect subsystem modules indicated in Figure 7, see the red lines and dots. The noise #2 is also generated with the ASG but injected into the two modules of the noise subsystem, see in Figure 7 the blue lines. The measurements are performed with the six HFCT sensors and two acquisition units shown in Figure 2 and Figure 7. The voltage reference signal required for the measurements is generated with the ASG, see the green lines in Figure 7. With the results obtained, Table 6 is completed. In this table it is indicated if the measuring system, in automatic mode, is able to report the presence of PD activity and to trigger any alarm. In addition, if it is able to report about: the number of defects, the phase or phases affected, the identification and location of the defects and the identification of the affected element.
- Realization of a second measurement for the evaluation of the capability to automatically analyze the evolution of defects over time. In this second test the capability to generate an alarm when critical levels of charge Q_IEC_ or PD rate are reached is also evaluated. In this test, a 30 min measurement is performed generating at least the three defects of the previous test (#2, #3, #4) with the two noises (#2 and #3), plus the additional aleatory noise #1 shown in Figure 6a,b. In this case, the aging of defect #2 is simulated, varying in 5 intervals of 6 min its Q_IEC_ and PD rate values. The values shown for each time interval in Table 3 are equivalent to the average of those measured in 200 h of aging of a real internal defect. To simulate on-site measuring conditions, the noise signals generation vary over time. The variation of these signals is performed by the generation of the successive combinations shown in the last row of Table 3.

An example of these tests’ realization is shown in Section 3.2. To show the results, the use of Tables 6 and 7 is recommended. 

**Table 2:** Defect and noise characteristics.

Type of Defect or Noise	Position	Affected Phase	Defective Element	Q_IEC_ (pC) or 3σ	PD Rate (ppp)	Test Where It Is Used
Defect #1 internal cavity	At the end of the line	-	Cable terminal	500–550	50–60	2
Defect #2 internal cavity	At the beginning of the line	R	GIS	500 (**)	50 (**)	3, 4, 5, 6, 7, 8 and 9
Defect #3 internal surface	At the beginning of the line	R	Cable terminal	500	50	3, 4, 5, 6, 7, 8 and 9
Defect #4 internal cavity	At the second joint	S	Second joint	500	50	3, 4, 5, 6, 7, 8 and 9
Noise #1 aleatory	In the noise subsystem	R-S-T	-	3.8 mV (*)	-	2, 3, 4, 5, 6, 7, 8 and 9
Noise #2 aleatory	In the noise subsystems	R-S-T	-	3.8 mV (*)	-	2,3, 4, 5, 6, 7, 8 and 9
Noise #3 pulse-type	At the beginning of the line	R-S-T	-	3.8 mV	-	3, 4, 5, 6, 7, 8 and 9

(*) In test 2, the level of this noise is adjusted in 3σ = 3.8 mV, 3σ = 7.6 mV, 3σ = 11.4 mV and 3σ = 15.2 mV. (**) In the second part of test 3, this value changes over time as shown in Table 3.

**Table 3:** Defect #2 characteristics and noise signals for the evaluation of the capability to analyze automatically the defect evolution over time.

Injected Signals (*)	Time (min)									
	0–6		6–12		12–18		18–24		24–30	
	Q_IEC_ (pC)	PD Rate (ppp)	Q_IEC_ (pC)	PD Rate (ppp)	Q_IEC_ (pC)	PD Rate (ppp)	Q_IEC_ (pC)	PD Rate (ppp)	Q_IEC_ (pC)	PD Rate (ppp)
Defect #2	520–550	55–60	490–520	50–55	450–490	45–50	410–450	40–45	380–410	35–40
Noises #1, #2 and #3	#2 (2 min)		#2 (2 min)		#2 (2 min)		#2 (2 min)		#2 (2 min)	
	#2 + #3 (2 min)		#2 + #3 (2 min)		#2 + #3 (2 min)		#2 + #3 (2 min)		#2 + #3 (2 min)	
	#1 + #2 + #3 (2 min)		#1 + #2 + #3 (2 min)		#1 + #2 + #3 (2 min)		#1 + #2 + #3 (2 min)		#1 + #2 + #3 (2 min)	

(*) In this test defects #3 and #4 are also generated.

**Test 4. Identification of the phase affected by a defect.** In HV electrical systems when PD activity occurs in a defect positioned in one phase, usually this activity can be detected in the other two due to the crosstalking phenomena. Thus, on some occasions the identification of the phase affected by a defect is not immediate. In this test, the capability of a measuring instrument to detect the phase affected by a defect is evaluated. 

To perform this test and the rest presented below, again the defects #2, #3 and #4 generated in the positions indicated in Table 2 and Figure 7 must be measured, along with the noises #2 and #3. For all tests, this proposal can be varied by considering the combination of other defects and noises in a greater or lower quantity and by performing (as in test 2) a progressive scaling of the noise signals. Examples of how to conduct this test and all the following ones, together with the results obtained, are presented in Section 3.2.

**Test 5. Number of defects determination.** In on-line measurements, as all assets are connected in the supervised installation, the presence of more than one PD source and pulse-type noise signals is quite likely. In these cases, pulses from various sources are superimposed in the phase resolved PD (PRPD) patterns. The consequences are that some defects may be hidden and not detected, or that the interpretation of the PRPD pattern does not allow the correct identification of the defects. In this regard, for the detection of all the defects, it is necessary to obtain individual PRPD patterns for each one. The results obtained in the previous test (identification of the phase affected) and in the following one (localization of defects) are useful to obtain individual patterns. In addition, there are also complementary diagnostic tools effective for the improvement in the separation and detection of defects. These tools are usually based on the analysis of the recorded pulse waveform [7,8,9]. The pulses generated in a defect have a similar waveform and generally, when they are measured, it differs from the waveforms of the pulses generated in other defects. In this test, the capability of the systems to detect and separate PD generated in various sources is assessed.

**Test 6. Localization of defects.** In HV electrical installations when PD activity occurs in an insulation defect the pulses propagate through the assets. Thus, considering the pulses’ speed in the propagation paths and the position of the measuring points, the location of PD sources is possible. When off-line tests are performed in cable systems, the reflectometry technique is commonly used, measuring phase by phase at one of the line ends. However, this technique is not recommended for on-line monitoring applications, since the impedance changes in the supervised installations are minor (all assets are connected), and consequently, the capacity to detect the PD source emplacement is very low. In on-line applications, the analysis of the time delay between the arrival times of PD pulses to consecutive sensors is commonly used for localization purposes. With this last technique the effectiveness in the location improves considerably. In this test the capacity of the systems to locate PD sources is assessed.

**Test 7. Identification of the type of defect.** PRPD patterns are related to the type of insulation defects [8,24], therefore in most cases by analyzing them defect identification is possible [25,26]. The recorded pulses generally correspond to a pulse-type noise signal, internal defect, surface defect, floating potential element or corona effect [9,24]. Several developments are focused on the mathematical and artificial intelligence processing of the information related to the PRPD patterns in order to automatically identify the defects associated with them [27,28]. Some of these developments are integrated in commercial measuring systems. In this test, the functionality of the systems to associate PRPD patterns to the corresponding defects is characterized. For an adequate identification of defects, each pattern to be analyzed should correspond to a single PD source. The individual patterns obtained in test 5 are processed by automatic diagnostic tools and the degree of accuracy in the identification of each defect is checked. The patterns to be identified in this test were previously validated by five independent expert analysts, and in all cases they unanimously agreed with the associated type of defect. The accuracy in the identification depends on the overlapped noise signal level. When the noise is more severe, the probability of obtaining a correct result decreases.

**Test 8. Identification of the defective element.** To perform complete diagnoses, the identification of the defective elements in the monitored installations is necessary, which helps to make appropriate decisions on the possible execution of corrective actions. The information gathered from the functionalities used for detecting, locating and identifying the defects is very useful for the identification of the defective elements. In addition, the discrimination of the affected element can be improved or corroborated by additional explorations, for example, by the polarity analysis of the pulses measured. In this test, the capability of PD systems to identify the defective elements is characterized. The defects detected and identified in the previous test, once associated with each PD source, must be assigned to a specific element (cable, terminal, joint or GIS compartment) and the degree of success is checked for each case. In the realization of this test, for a general overview and analysis of the results the use of Table 11 is recommended.

**Test 9. Determination of the Q_IEC_ and PD rate values.** In PD measurement, to perform accurate and complete diagnoses, the quantification and control of the defect evolution over time is very important. This consideration was taken into account in the definition of test 3 (autonomous diagnosis and alarm management). The reference magnitude for the quantification of PD activity is the apparent charge, which is measured according to the reference standard IEC 60270 [29,30]. To determine the charge of a set of PDs associated with an individual PRPD pattern, the value of the largest repetitive PD in a considered time interval is quantified. The charge value of the set is known as Q_IEC_. On the other hand, PD activity is also quantified by the pulses’ repetition rate. For the obtention of the Q_IEC_ value according to IEC 60270, a previous calibration of the measurement system is required. The acquisitions are realized with a coupling capacitor and quadrupole, measuring in frequency ranges below 1 MHz. This measuring technique, suitable to perform tests in shielded laboratories, is not suitable for on-site and on-line measurements for the following three reasons.

- It is an invasive technique, except when the measurements are performed in the capacitive tap of power transformer bushings.
- The measurements are performed in frequency ranges where noise rejection is often challenging.
- For the bandwidths specified, although the resulting charge value is obtained conveniently and matches the integral of the current pulse in the time domain, the waveform of the pulses is lost. Thus, if the original signals are not accessible, some diagnostic tools such as those used for PD source separation by the pulse waveform analysis cannot be applied.

For the above reasons, in on-site and on-line measurements non-invasive techniques are applied according to the technical specification IEC 62478 [1], measuring in frequencies above 1 MHz. Under these conditions, the Q_IEC_ value is generally difficult to quantify. For its estimation, the performance of a previous off-line calibration is required to know the pulses’ behavior in their propagation medium and the transfer function of the sensors used. On the other hand, for the estimation of the Q_IEC_ value, the capability of the systems to analyze the pulses’ waveform in the time and frequency domain is very useful. In addition, the systems can incorporate a functionality for the analysis of the PD pulses’ attenuation. It is worth noting that the information gathered after applying the separation and localization functionalities is essential for the estimation of the Q_IEC_ value of each defect.

The other important parameter for the diagnosis is the PD repetition rate, since for certain defects this parameter is associated with their degree of criticality. Furthermore, the analysis of the Q_IEC_ and PD rate values’ evolution over time is also important to estimate the criticality of defects. 

In this test the capability of the measuring systems to determine the Q_IEC_ and PD rate values is characterized. A Q_IEC_ value must be assigned to each of the defects detected in the previous tests, using the results of the off-line calibration performed in test 2 (noise rejection) and of the functionalities of location, pulse analysis in the time and frequency domain, attenuation or others. A PD rate value must also be assigned to each defect. The reference Q_IEC_ and PD rate values of the defects generated are specified in Table 2. The degree of success obtained for these two parameters is analyzed. For a general overview of the results the use of Table 12 is recommended.

## 3. PD Measuring System Characterization

For a deeper understanding of the method and with the aim of showing the convenience and advantages of its implementation in the test platform, an applicability example is presented in this section. The proposed case study is focused on the characterization of the functionalities of a commercial PD monitoring system, mainly developed for the on-line supervision of HV electrical installations. 

### 3.1. Measuring System Characteristics and Functionalities

The main technical characteristics of the PD measuring system concerning the acquisition units are: bandwidth 60 MHz, sample rate 125 MS/s, vertical resolution 14 bits and input impedance 50 Ω. For the determination of the defect location, the acquisition units can be synchronized to the same time reference in two ways: when they are positioned in the same emplacement (for example, in the same HV substation), by means of a fiber optic cable, and when they are in different emplacements by GPS [9]. For the measurements, HFCT sensors with a bandwidth from 100 kHz to 20 MHz were used. 

For test 2 (noise rejection), this measuring system has an automatic filtering tool based on the wavelet transform. With this filter, pulse-shaped signals with amplitudes even below the noise level can be detected. However, not all the pulses detected correspond to PD activity, as pulse-type disturbances cannot be discriminated. Nevertheless, the application of additional functionalities such as those used in the tests “identification of the phase affected”, “number of defects determination” or “localization of defects” enables the discrimination of pulse-type electrical noise from PD activity. 

For test 3 (autonomous diagnosis and alarm management), the system has an automatic diagnostic functionality based on management software that processes the data obtained with the functionalities used in tests 4, 5, 6, 7, 8 and 9. With this functionality, trend graphics of the Q_IEC_ and PD repetition rate values, obtained for the defects under supervision, can be automatically analyzed.

For test 4 (identification of the phase affected by a defect), the system has a functionality with which the pulses measured at the same instant by the three sensors of each measuring point are analyzed. For each set of pulses recorded in the three phases, their charge is quantified and the values are plotted in a 3D diagram. The analysis of the clusters formed makes the detection of the phases affected by defects possible. 

For test 5 (number of defects determination), a functionality for the separation of coexisting defects and pulse-type noise signals known as a “clustering tool” is used. This functionality is based on the analysis of three parameters, two related to the individual pulse waveform and the third to their frequency spectrum [9]. The values of these parameters are represented in a 3D diagram, where the defects can be differentiated by selecting the formed clusters.

For test 6 (localization of defects), there is a defect location functionality with which the arrival times of the pulses at the sensors are analyzed. To determine the location of the defects, the distance between the measurement points and the pulse propagation speed in their propagation medium are considered [9]. The PD source location is visualized in a mapping diagram.

For test 7 (identification of the type of defect), the system has a functionality for the automatic identification of defects based on an artificial neural network approach. This functionality is developed in a data model based on a convolutional neural network. Once the individual patterns are analyzed, in the identification results the associated hit probability and the criticality of the defects are indicated.

For test 8 (identification of the defective element), with this system, the information gathered in the detection, location and identification of the defects is analyzed. Furthermore, an additional functionality with which the polarity of the measured pulses is also analyzed is used. The polarity analysis makes it possible to determine whether the defective element is located on one side or the other of the measuring point [9]. The combined analysis of the previous results is useful for the identification of the defective element of the installation.

For test 9 (determination of the Q_IEC_ and PD rate values), the information gathered with the detection and location functionalities is considered for the estimation of these values. In addition, for the estimation of the Q_IEC_ values, the pulses are analyzed in the time and frequency domain and an additional functionality for the analysis of the signal attenuation in the propagation medium is applied.

### 3.2. Method Application to Perform the Characterizations

In steps 1 and 2 of stage 1 of the method (see Figure 3), the test platform shown in Figure 2 and Figure 7 was assembled and set. For the commissioning of the measuring system (step 3) the following setting parameters were considered: distance between measuring points (1740 m) and pulse propagation speed (1164.2 m/µs) [18]. Once the measuring system was commissioned, everything was ready for the test realization. In stage 2 of the method the characterization tests were set. In step 4, all the reference tests listed in Table 1 were considered. In step 5 the subsystems were adjusted. The defects and noises that were generated for the tests in steps 6_a and 6_b, respectively, along with the quantities to be adjusted in step 7, are specified in Table 2 and Table 3. In stage 3 of the method the measuring system functionalities were characterized. The PD measurements were performed, the data were recorded and processed and the results were analyzed (step 8). 

The results obtained for each test are shown below.

**Test 1. Sensitivity in the detection.** This test is completed by checking the linearity of the measuring system scale factor. To avoid the effects of background noise, for each charge level, the average value of the synchronized samples of 500 captures was calculated. The results obtained are shown in Table 4. Under the conditions in which the test was performed, with the sensor and acquisition unit to be characterized, the system was no longer sensitive when pulses lower than 10 pC were injected. For 10 pC, the error in the measurement was 10%. The sensitivity achieved can be considered very satisfactory, as pulses below 10 pC were detected. 

This test and all the following ones can be repeated for another HFCT sensor and/or acquisition unit for the purpose of performing comparisons among various technologies.

**Test 2. Noise rejection.** This test was conducted with level 2 of difficulty. Analyzing the results shown in Table 5, the detection of the defect was not clear when the injected noise was 3σ = 11.4 mV. With this noise level, the error percentages for the Q_IEC_ and PD rate were 89.3% and 82.1%, respectively. For a further characterization, this test can be repeated by injecting another defect and/or noise characteristic of real installations. In the PRPD pattern measured with this noise level, see the last row of Table 5, very few PD pulses were detected in the positive half-cycle of the reference voltage signal.

**Test 3. Autonomous diagnosis and alarm management.** In this test, with the measurements of the three defects together with the noises, the results shown in Table 6 and Table 7 were obtained. Analyzing these results, it can be stated that with this measuring system, the realization of diagnosis and the generation of alarms in automatic mode are possible, see Table 6. Furthermore, with this system, the supervision of the defect evolution over time in automatic mode is also possible, see in Table 7 the results analyzed in each time interval for the Q_IEC_ and PD rate values. It can be observed that an alarm was triggered in the time interval between minutes 18 and 24.

**Figure 8:**
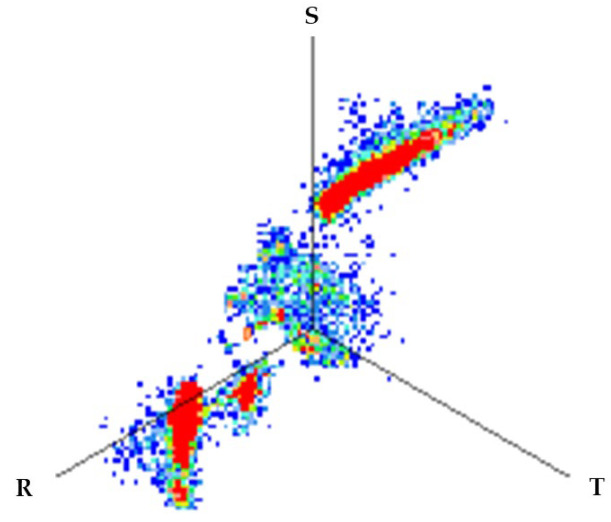
The 3D diagram of the charge values obtained for PD measured simultaneously with the three sensors positioned at the beginning of the distribution line.

**Test 4. Identification of the phase affected by a defect.** The result obtained for the measurements performed with the three sensors positioned at the beginning of the distribution line is shown in Figure 8. In this 3D diagram, three or four clusters of pulses can be observed, one or two in phase R (it is not clear), one in phase S and one in the center. With the result obtained it can be indicated that the phases affected by possible defects are R (probably with two defects) and S (probably with one). The cluster in the center can be generally associated with pulse-type noise signals.

**Test 5. Number of defects determination.** In this test the clustering tool of the measuring system is applied to the pulses of the patterns obtained in the measurements performed with the six sensors. The results shown in Table 8 were obtained for the measurements performed at the beginning of the distribution line in phases R and S. For these two phases, the raw patterns, the 3D clustering diagrams and individual patterns associated with each cluster are displayed.

In phase R, three clusters (#1, #2 and #3) were detected. Analyzing their respective patterns shown in the last row, it can be stated that clusters #1 and #2 correspond to the insulation defects #2 and #3, respectively, and cluster #3 corresponds to the pulse-type noise #3. In phase S, two clusters were detected (#4 and #5). Analyzing their patterns, it can be stated that cluster #4 corresponds to the insulation defect #4 and cluster #5 to the pulse-type noise #3. It can be observed that, with the analysis performed, the three defects generated were detected. This result can be reinforced or corroborated with the result obtained in the identification of the phase affected test, where the presence of one or two defects in phase R and one in phase S was intuited, see Figure 8.

**Test 6. Localization of defects.** The results obtained for the defect localization are shown in the first row of Table 9. With these results, it can be indicated that there is at least one defect in phase R positioned at the beginning of the line. The defect/s in this phase can be either in the GIS or in the cable terminal. In addition, it is possible to indicate that there is at least one defect in phase S, positioned in the second joint at 1160 m. This test was completed with the analysis of the PRPD patterns associated with each location. By combining the defect location functionality and the clustering tool, the discrimination of defects located at the same position is possible, see the last two rows of Table 9. For phase R, the defects #1 and #2 located at the same site (corresponding to clusters #1 and #2) were discriminated and corroborated. The presence of the single defect #3 in phase S was also corroborated.

**Test 7. Identification of the type of defect.** With this test, the capability of the measuring system to automatically identify the defects is characterized. The starting points are the individual PRPD patterns obtained in tests 5 and 6, see Table 8 and Table 9. These patterns are associated in automatic mode with the defect types that generate the PD activity. Applying the defect identification functionality of the measuring system to the individual patterns, the results shown in Table 10 were obtained. For patterns #1 and #4 an internal cavity-type defect was identified, for pattern #2 an internal surface-type defect and for patterns #3 and #5 a pulse-type noise. The criticality associated with the defects was high.

**Test 8. Identification of the defective element.** The information considered in this test for the defective element identification is shown in Table 11. In this table the pulse polarity, individual PRPD patterns, types of defect and emplacements are indicated. The result of the defective element identification is shown in the last row.

The row of the pulse polarity, see Table 11, shows the waveform of a representative pulse of each pattern, measured in the positive half cycle of the reference voltage signal. Analyzing the results, with regard to defects #2 and #3 of phase R, with the type of defect and emplacement information it is not possible to know whether these defects are in the GIS compartment or in the cable terminal. However, as the polarity of the pulses is negative for the pattern of defect #2 and positive for the pattern of defect #3, it can be stated that defect #2 is generated in the GIS and #3 in the cable terminal. On the other hand, the defect of phase S is in the cable joint located at 1160 m, since PD activity was located at an intermediate point between the sensor positions [9]. In this test, all the elements affected by a defect were identified.

**Test 9. Determination of the Q_IEC_ and PD rate values.** With this test, the system capabilities to estimate the Q_IEC_ and PD rate values for each defect are evaluated. The results obtained are shown in Table 12.

The most accurate result of Q_IEC_ was obtained for the internal defect #3, which was positioned in the cable terminal of phase R. It should be noted that it was at this point where the calibration of the system was performed. To determine the Q_IEC_ value of defect #4, which was positioned in the second joint of phase S, it was necessary to use the functionality of the measuring system that enables the estimation of the pulse attenuation with distance. The most accurate result of PD rate was also obtained for the defect #3.

## 4. Conclusions

For technology developers and users of PD measuring systems, the possibility of implementing a reference method for their characterization in a complete way is of utmost importance. The most effective systems will enable the realization of highly accurate diagnosis in a less costly way. With the proposed method in this research and its implementation in the associated test platform that simulates an HV insulated distribution line, a solution is offered to these companies, given the lack of a comprehensive reference method and the technical and economic difficulties encountered when characterizing PD measuring systems. In particular, the solution presented is focused on the characterization of systems that measure on-line and with HFCT sensors.

The presented characterization of a commercial system has been useful to show the validity of the method and of its implementation in the test platform, demonstrating the advantages of their combined use. 

With the development carried out in this research, it is possible not only to characterize PD systems measuring on-line and with HFCT sensors but also to perform comparisons among them in a convenient and user-friendly way. 

Although the method and platform are designed for the characterization of systems operating on-line and using HFCT sensors, both could be adapted in an evolved version to carry out characterizations of systems operating off-line or with other types of sensors. On the other hand, the test platform can be extended to a more complex one, in which more elements of HV installations can be simulated and, thus, the characterizations can be performed with various scenarios and simulating other defects and noises.

The realization of the tests indicated in the method using the platform is currently serving to characterize not only the functionalities of PD measuring systems but also the capabilities of analyst technicians. In addition, the method–platform set is being used to carry out training tasks for electrical engineering students, new researchers and technicians of electrical companies. Furthermore, technology developers are using the method and platform to perform practical demonstrations, showing to their potential customers the effectiveness and advantages of using their technology.

With this new method applied in the test platform, the previous tasks (characterization, training and demonstration) can be performed everywhere and anytime due to the portability and technical reproducibility of the test platform. In addition, all these tasks can be carried out without the requirement of applying HV.

## 5. Patent

The developments related to the method and test platform presented in this research article are protected by patent application no. P-202331099 and reference no. P-102092, filed with the Spanish Patent and Trademark Office.

## Figures and Tables

**Table 1:** Reference tests for the characterizations.

Test	Name	Test	Name
1	Sensitivity in the detection.	6	Localization of defects.
2	Noise rejection.	7	Identification of the type of defect.
3	Autonomous diagnosis and alarm management.	8	Identification of the defective element.
4	Identification of the phase affected.	9	Determination of the Q_IEC_ and PD repetition rate values.
5	Number of defects determination.		

**Table 4:** Results of the sensitivity in the detection test.

Injected Pulse Charge (pC)	Sensitivity (Pulses Detected?)	Measured Values and Errors	
		Pulse Charge (pC)	Error (%)
1000	Yes	1000 (scale factor = 542.6)	-
500	Yes	502	0.4
200	Yes	203	1.5
100	Yes	103	3
50	Yes	52	4
20	Yes	21	5
15	Yes	16	6.7
10	Yes	11	10
9	Yes	11	22.2
8	No	-	-

**Table 5:** Results of the noise rejection test.

Injected Noise Signal		Background Base Noise + Noise #2 (3σ = 3.8 mV)	Background Base Noise + Noise #2 (3σ = 7.6 mV)	Background Base Noise + Noise #2 (3σ = 11.4 mV)	Background Base Noise + Noise #2 (3σ = 15.2 mV)
Q_IEC_ value	Injected (*) 500–550 (pC)	545	527	506	550
	Measured (pC)	539	172	54	5
	Error (%)	1.1	67.4	89.3	99.1
PD rate	Injected (*) 50–60 (ppp)	58	52	56	55
	Measured (ppp)	56	24	10	1
	Error (%)	3.4	53.8	82.1	98.2
PRPD pattern		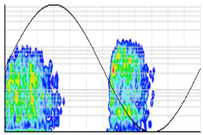	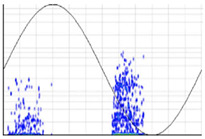	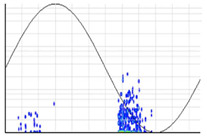	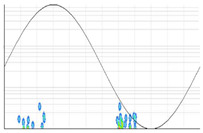

(*) The values in these rows are randomly generated and only known to the assessor.

**Table 6:** Results of the autonomous diagnosis and alarm management test.

Presence of PD Activity?	Alarms Triggered?	Number of Defects Detected	Affected Phases	Type of Defects Identified	Defect Location	Affected Elements
Yes	3	3	R and S	Internal cavity	0 m	-
				Internal surface	0 m	Cable terminal
				Internal cavity	1164 m	Cable joint

**Table 7:** Supervision of the defect #2 evolution over time and automatic alarm generation.

Time Interval (min)	0–6	6–12	12–18	18–24	24–30
Alarm detected?	No	No	No	Yes	Yes
Alarm triggered time	-	-	-	19′06″	-
Q_IEC_ trend	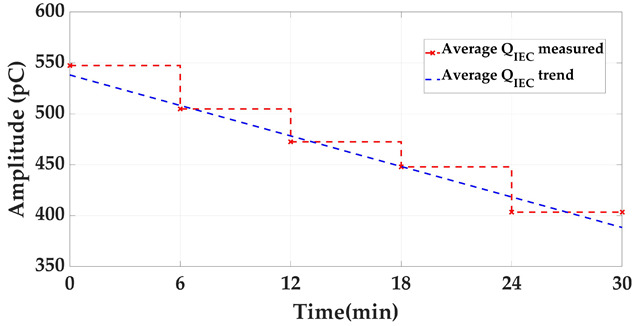				
PD rate trend	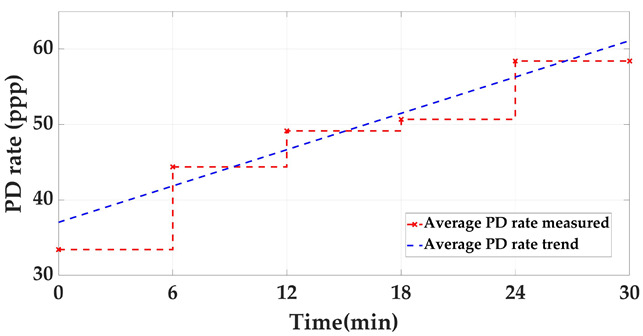				

**Table 8:** Results of the number of defects determination test.

**Raw PRPD patterns obtained in one position and phase**	**Measurement in phase R at the beginning of the line**	**Measurement in phase S at the beginning of the line**
	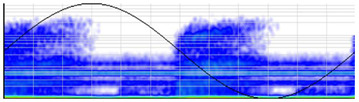	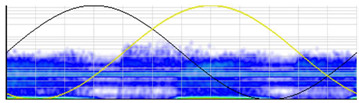
**3D clustering diagram**	**3D diagram obtained for phase R**	**3D diagram obtained for phase S**
	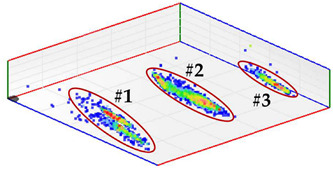	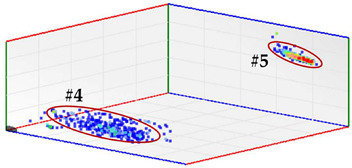
**Individual PRPD patterns per cluster**	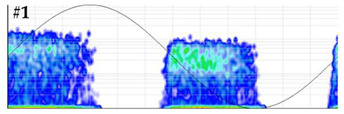	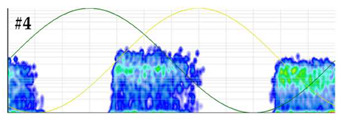
	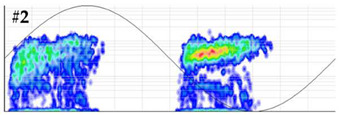	
	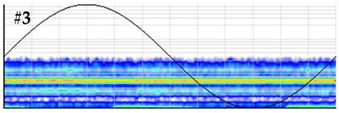	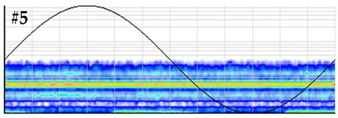

**Table 9:** Results of the defect localization test and discrimination of defects located at the same emplacement.

**PD source mapping diagram**	**Phase R**	**Phase S**
	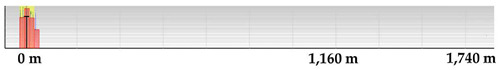	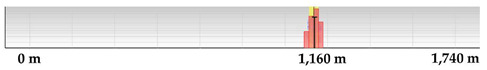
**3D clustering diagram per location**	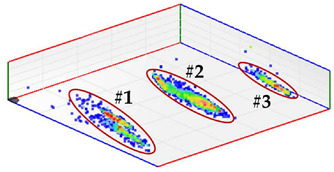	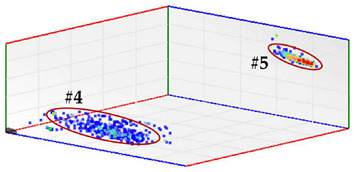
**Individual PRPD patterns for clusters #1, #2 and #3**	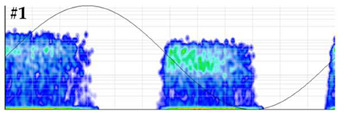	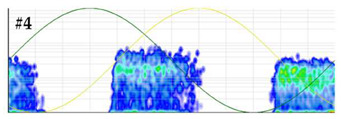
	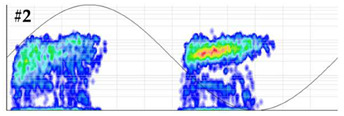	

**Table 10:** Results of the identification of the type of defect test.

**Pattern #1 (Defect #2)**	**Pattern #2 (Defect #3)**
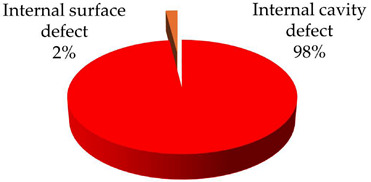	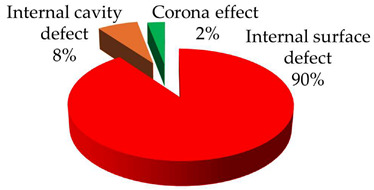
**Pattern #4 (Defect #4)**	**Patterns #3 & #5**
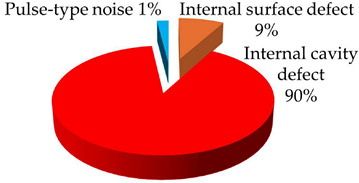	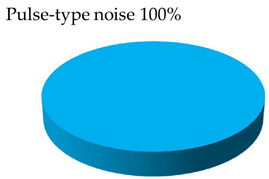

**Table 11:** Results of the identification of the defective element test.

Defect	#2	#3	#4
Pulse polarity	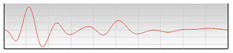	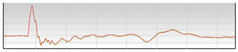	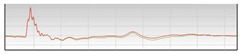
PRPD patterns	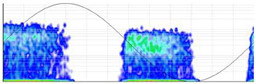	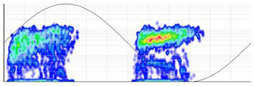	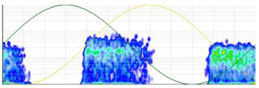
Type of defect	Internal cavity	Internal surface	Internal cavity
Emplacement	At the beginning of the line in phase R	At the beginning of the line in phase R	In the second joint in phase S
Defective element	GIS	Cable terminal	Cable joint

**Table 12:** Results of the test performed for the determination of the Q_IEC_ and PD rate values.

Defect	Type	Q_IEC_ Value			PD Rate		
		Injected	Measured	Error (%)	Injected	Measured	Error (%)
#2	Internal cavity	500	479	4.2	50	47	6
#3	Internal surface	500	491	1.8	50	49	2
#4	Internal cavity	500	487	2.6	50	45	10

## Data Availability

Data are contained within the article.
